# Establishment of a rapid diagnosis method for *Candida glabrata* based on the *ITS2* gene using recombinase polymerase amplification combined with lateral flow strips

**DOI:** 10.3389/fcimb.2022.953302

**Published:** 2022-07-28

**Authors:** Kun Wang, Li Huo, Yuanyuan Li, Lihua Zhu, Yan Wang, Lei Wang

**Affiliations:** ^1^ Department of Medicine Laboratory, Second People’s Hospital of Lianyungang (Cancer Hospital of Lianyungang), Lianyungang, China; ^2^ Department of Laboratory Medicine, Affiliated Hospital of Jiangsu University, Zhenjiang, China; ^3^ School of Biotechnology, Jiangsu University of Science and Technology, Zhenjiang, China

**Keywords:** *Candida glabrata*, recombinase polymerase amplification, lateral flow strip, ITS2, qPCR

## Abstract

*Candida glabrata* is the second or third most common Candida-associated species isolated from hospital-acquired infections, surpassing even *C. albicans* in some hospitals. With the rapid progression of the disease course of *C. glabrata* infections, there is an urgent need for a rapid and sensitive on-site assay for clinical diagnosis. Isothermal amplification is a recently developed method for rapid nucleic acid detection that is being increasingly used for on-site detection, especially recombinase polymerase amplification (RPA). RPA combined with lateral flow strips (LFS) can rapidly amplify and visually detect the target gene within 20 min. The whole detection process can be controlled within 30–60 min by rapid sample pre-treatment. In this study, RPA-LFS was used to amplify the *internal transcribed spacer region 2* gene of *C. glabrata*. The primer–probe design was optimized by introducing base mismatches (probe modification of one base) to obtain a highly specific and sensitive primer–probe combination for clinical sample detection. RPA-LFS was performed on 23 common clinical pathogens to determine the specificity of the assay system. The RPA-LFS system specifically detected *C. glabrata* without cross-reaction with other fungi or bacteria. Gradient dilutions of the template were tested to explore the lower limit of detection of this detection system and to determine the sensitivity of the assay. The sensitivity was 10 CFU/µL, without interference from genomic DNA of other species. The RPA-LFS and qPCR assays were performed on 227 clinical samples to evaluate the detection performance of the RPA-LFS system. Eighty-five samples were identified as *C. glabrata*, representing a detection rate of 37.5%. The results were consistent with qPCR and conventional culture methods. The collective findings indicate a reliable molecular diagnostic method for the detection of *C. glabrata*, and to meet the urgent need for rapid, specific, sensitive, and portable clinical field-testing.

## Introduction

*Candida* spp. fungi are the most common opportunistic pathogens. The five most common are *Candida albicans, C. glabrata, C. parapsilosis, C. tropicalis*, and *C. krusei*, which account for 95% of human pathogenic fungal infections (Sendid et al., 2002; Khodadadi et al., 2017). Although *C. albicans* is still the most common fungus in clinical practice, its isolation rate has decreased in recent years. Compared to non-*C. albicans*, infections caused by *C. glabrata* have the highest morbidity and mortality rates, ranging from 40–70% (Krcmery and Barnes, 2002). The morbidity and mortality rates of *C. glabrata* occurring in oncology patients and bone marrow transplant patients are as high as 50% and 100%, respectively (Li et al., 2007). *C. glabrata* has become the second or third most common pathogen. The number of mucosal and systemic infections caused by *C. glabrata* has increased significantly. *C. glabrata* can cause infection alone or along with other pathogenic fungi, such as *C. albicans*, and there are no specific clinical symptoms (Tati et al., 2016). Therefore, a rapid and accurate diagnostic method for *C. glabrata* has become the key to the prevention and treatment of candidiasis.

At present, identification of *C. glabrata* typically uses the traditional fungal culture method. This approach is time-consuming and cumbersome, which limits the early diagnosis of *C. glabrata* and delays effective treatment. Highly sensitive and specific molecular biology methods used for *C. glabrata* include polymerase chain reaction (PCR) restriction fragment length polymorphism (PCR-RFLP) based on DNA amplification (Neji et al., 2017), real-time PCR (Souza et al., 2012), random amplified polymorphic DNA (RAPD) (Al-Tekreeti et al., 2018), PCR analysis of polymorphism by intron length, matrix-assisted laser desorption ionization time-of-flight mass spectrometry (MALDI-TOF MS) (Ferreira et al., 2011), sequencing analysis of pan-fungal markers, and isothermal assays (Guinea et al., 2021). The main isothermal amplification techniques are the loop-mediated isothermal amplification (LAMP) assay (Fallahi et al., 2020), rolling circle DNA amplification (Carinelli et al., 2017), nuclear acid sequence-based amplification (Huang et al., 2019), single primer isothermal amplification (Yang et al., 2021), isothermal amplification dependent on helicase-dependent isothermal DNA amplification (HAD) (Jeong et al., 2009), and strand displaced amplification (Zhang et al., 2017). Recombinase polymerase amplification (RPA) is a recently developed thermostatic amplification technique that can combine the advantages of the above methods and compensate for their shortcomings, which could potentially be valuable as a rapid, specific, sensitive, and portable diagnostic assay. We previously reported a related study on the detection of *C. albicans* using an RPA-lateral flow strip (LFS) protocol, which provides a reference for the detection of *C. glabrata* (Wang et al., 2021).

The specific RPA reaction is constructed using the *internal transcribed spacer protein-2* (*ITS2*) gene of *C. glabrata* as the detection target. The amplification product is labeled with fluorescein isothiocynate (FITC), which permits effective and accurate color development through the lateral flow strips (LFS) (Piepenburg et al., 2006). The assay process is simple and rapid. The reaction time at 37°C is approximately 20 min, and the LFS results can be observed in only 5 min. Because the assay system is less dependent on the laboratory environment, LFS is suitable for on-site accurate diagnosis of diseases. In addition, the assay system can be extended to different areas, such as healthcare and food safety, and used to detect other pathogenic bacteria from different sources. This is important to promote the development of clinical testing and diagnostics and its interdisciplinary aspects.

In this investigation, we obtained primer–probe sets with high specificity and sensitivity by carefully modifying the primer and probe bases. The results obtained using these sets were compared with the experimental results of qPCR and conventional culture methods by testing clinical samples. The aim was to ensure that our established method could be used for the diagnosis of Candida infections caused by *C. glabrata*.

## Materials and methods

### Ethics statement

The study protocol was approved by the Medical Ethics Committee of the Second People’s Hospital of Lianyungang City (Lianyungang, Jiangsu, China) (permit number 2020013). Informed consent was obtained from patients prior to collection of clinical samples.

### Strain acquisition

*C. glabrata* ATCC 15126/66032/2001/64677 was purchased from Shanghai Covey Chemical Technology Co., Ltd. (Shanghai, China). Twenty strains of *C. glabrata* were isolated from clinical samples collected from 2020 to 2021. The specificity of the RPA-LFS assay was verified based on the *ITS2* gene (GenBank: MF769562.1) of 23 common pathogens stored in our laboratory. These included *C. parapsilosis* ATCC 22019, *C. krusei* ATCC 14243, *C. tropicalis* ATCC 20962, *C. albicans* ATCC 10231, *C. auris, C. dubliniensis, C. neoformans* ATCC 14116, *Acinetobacter baumannii* ATCC 19606, *A. calcoaceticus, A. lwoffi, A. haemolytius, A. junii, Aspergillus fumigatus, Enterococcus faecium, Escherichia coli* O157, *Staphylococcus aureus*, *S. capitis*, *S. epidermidis*, *S. haemolyticus*, *S. hominis*, *S. saprophyticus*, *S. warneri*, and *S. maltophilia*. In total, 227 clinical samples suspected of fungal infection were collected and pre-processed by the microbiology team of our laboratory in our hospital. The samples included 121 blood, 53 sputum, 35 urine, and 18 other samples.

### Genomic DNA extraction

All bacterial strains were boiled at 100°C for 10 min before being used as templates for DNA release. Unless otherwise stated, 1 μL 10^5^ colony forming units (CFU)/mL heat-treated cultures were used as templates. For *C. glabrata* and other fungi, genomic DNA was extracted and purified from cultures or clinical samples using the GeneJET Genomic DNA Purification Kit (Thermo Fisher Scientific, Waltham, MA, USA) according to the manufacturer’s instructions. An Invitrogen™ Qubit™ 4 Fluorometer (Thermo Fisher Scientific) was used according to the manufacturer’s instructions to quantify the extracted genomic DNA.

### Primer and probe design and screening

Two pairs of RPA primers based on the *ITS2* gene were designed using Primer Premier 5.0 software (Premier Biosoft International, San Francisco, CA, USA). After entering the sequence of a specific target region, the primers parameters were set as follows: product size of 80–150 bp and primer size of 30–35 bp, with no more than three consecutive bases in the three ends of complementary pairing. The maximum hairpin score, maximum primer-dimer score, and maximum poly-X score were each 5. The species specificity of the primer and probe sequences were confirmed using Primer-BLAST on the NCBI website (https://www.ncbi.nlm.nih.gov/tools/primer-blast). Forward primers were extended 15-23 bp backward at the 5’ ends and the performance of the probes and reverse primers was evaluated using Primer Premier 5 software. Theoretically, the formation of dimer and hairpin structures between the probe and the reverse primer was avoided as much as possible with a probe size of 46–53 bp, GC content of 30–80%, and Tm of 57–80. The 5’ end of the probe was labeled with FITC, the 3’ end was closed with a C3 spacer, and the middle base of the probe was replaced with tetrahydrofuran (THF). There were at least 30 bases before THF and then at least 15 bases following. The 5’ ends of the reverse primers were labeled with biotin.

### RPA reaction

RPA reactions were performed using the TwistAmp^®^ Liquid DNA Amplification Kit (TwistDx Inc., Maidenhead, UK) according to the manufacturer’s instructions. The 50 µL reaction system contained 25 µL 2× reaction buffer, 5 µL 10× Basic e-mix, 2.5 µL 20× core mix, 2.4 µL of 10 µM forward primer, 2.4 µL of 10 µM reverse primer, and 9.2 µL of distilled water. A 2.5 µL volume of 280 mM magnesium acetate and 1 µL of template were added to the lid of the reaction tube. After a brief centrifugation, the reaction mixture was incubated for 30 min at 37°C. RPA amplification products were purified by a PCR cleaning kit (Shanghai Meiji Biotechnology Co., Ltd., Shanghai, China) and used for 2% agarose gel electrophoresis.

### RPA-LFS assay

RPA reactions were performed according to the manufacturer’s instructions for the Twist Amp DNA amplification nfo kit (TwistDx Inc.). Each 50 μL volume of reaction mixture contained 2.1 μL of forward and reverse primers (10 μM), 0.6 μL of probe (10 μM), 2.0 μL of template, and other standard reaction components. Primers and probes were synthesized by Anhui General Biotechnology Co., Ltd. (Hefei, China). To start the reaction, 2.5 μL of magnesium acetate (280 mM) was added, and the reaction mixture was incubated at 37°C for 20 min. Then, 5 μL of the amplification product was diluted 20-fold and assayed by LFS (Usta Biotechnology Co., Ltd., Hangzhou, China). The LFS system consists of a sample pad, gold standard antibody pad impregnated with a mouse gold nanoparticle-labeled anti-FITC antibody, test line encapsulated with streptavidin, control line encapsulated with an anti-mouse antibody, and an absorption pad, arranged by solvent migration. The LFS was inserted into 100 μL of solvent for approximately 2 min until the test and control lines were visually detected.

### Conventional culture

The 227 clinical samples were incubated on Stachybotrys medium for 24–48 h and then further inoculated on Candida chromogenic medium. The initial identification was based on the color of the medium. *C. albicans*, *C. glabrata*, *C. tropicalis*, and *C. krusei* harbor enzymes interact with the substrate and produce green, purple, dark blue, and pink colors, respectively. Using the DL-96IImicrobiological assay system (Zhuhai Deer Biological Engineering Co., Ltd., Guangdong, China), the isolated and cultured fungi were identified according to genus and species. Each fungal suspension was added to the biochemical reaction wells and incubated at 28–30°C. The biochemical reactions directly produced the color changes due to fungal metabolism or color changes after the addition of chromogenic agents.

### Quantitative PCR analysis

Primers and probes are listed in **Table 1**. Specific primers and probes designed in our laboratory were targeted to the *ITS2* gene of *C. glabrata* for qPCR detection. The qPCR reaction mixture consisted of 12.5 μL MonAmp™ SYBR Green qPCR mixture (Mona Biotech, Suzhou, China), 0.5 μM forward and reverse primers, 1 μL genomic DNA, and distilled water added to 25 μL. The cycling program was 95°C for 10 min, followed by 40 cycles on a LightCycler 480 qPCR machine (Roche, Basel, Switzerland) at 95°C for 15 s and 55°C for 60 s, respectively.

**Table 1:** Primers and probes.

Primers/Probes	Primer Sequences	Size (bp)	Reaction name
*CG*-F1	TGGAGTTTACTTTACTACTATTCTTTTGTTCGTTG	35	RPA
*CG*-R1	TTGTTTTCTACTTGTTTCAATCTTGTGTTG	30	
*CG*-F2	GTGGAGTTTACTTTACTACTATTCTTTTGTTCGTT	35	
*CG*-R2	GTTGTTTTCTACTTGTTTCAATCTTGTGTT	31	
*CG*-P	FITC-GTGGAGTTTACTTTACTACTATTCTTTTGTTCGTT[THF]GGGGAGCGCTCTCTTT-C3 spacer	52	RPA-LFS
*CG*-R2B	Biotin-GTTGTTTTCTACTTGTTTCAATCTTGTGTT	31	
*CG*-F3	AGAGCAAACTGGGAAGGATCATTAAAGAAA	30	
*CG*-F4	TGCTGTGAATGCCATTTCTCCTGCCTGCGCTTAA	34	
*CG*-F5	CTGTGAATGCCATTTCTCCTGCCTGCGCTTAA	32	
*CG*-F6	GTGAATGCCATTTCTCCTGCCTGCGCTTAA	30	
*CG*-F7	GGGGAGGGAGCCGACAAAGACCTGGGAGTGT	31	
F	AGCAAACTGGGAAGG	15	qPCR
R	AGGCAGGAGAAATGG	15	

F, forward primer; R, reverse primer; P, probe.

## Results

### Primer validation screening strategy

**Figure 1:**
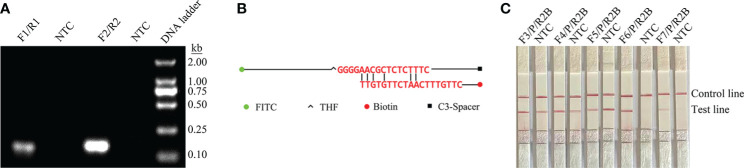
Screening of primer–probe combinations. **(A)** RPA results for two different primer sets of the *ITS2* gene. The name of each primer group is shown at the top of each lane. NTC indicates the no-template control for the corresponding primer pair. All reactions were performed at 37°C for 20 min. These images represent the results of three independent experiments. **(B)** Pairwise analysis and sequence modification of the primer–probe set were used to detect the *ITS2* gene using Primer Premier 5 software, and the associated DNA base substitutions for the probes and primers. The DNA strands are shown as horizontal lines and the matching bases are shown as vertical lines. Molecular markers are listed below in Figure **(B)**. **(C)** RPA-LFS assays for the validity of primer–probe sets. The name of each primer set is shown at the top of each lane. NTC denotes no-template control for the corresponding primer pair. The positions of the test and control lines are shown on the right. All reactions were performed at 37°C for 20 min. These images represent the results of three independent experiments.

*ITS2* was selected from the *C. glabrata* genome as a target for RPA-LFS detection. Two potential primer pairs were obtained by searching for primers on the *ITS2* gene sequence using NCBI primer-BLAST (**Table 1**). These primers were initially screened by target gene fragment amplification and no-template control. The amplification products were examined by agarose gel electrophoresis to compare the amplification performance of the target gene and primer-dimer formation in the no-template control. The F2/R2 primer pair was selected. This pair displayed the best amplification performance and no primer-dimer formation (**Figure 1A**). Candidate probes were obtained by extending the 3’ end of the forward primer F2 by 16 bp. All possible dimers generated by the probe and reverse primer were predicted, and then the bases were modified (**Table 1**) until no cross dimer could be formed (**Figure 1B**). Finally, five forward primers were designed, screened, and tested upstream of the probe. The LFS results showed that F3/R2/P, F4/R2/P, F5/R2/P, F6/R2/P, and F7/R2/P amplified the target gene fragment efficiently, but F5/R2/P amplified less efficiently, while F3/R2/P, F4/R2/P, and F5/R2/P without template negative controls all showed False-positive results, and only F6/R2/P met the detection requirements (**Figure 1C**). Therefore, F6/R2/P was used in subsequent experiments.

### Sensitivity determination of the PA-LFS assay system

**Figure 2:**
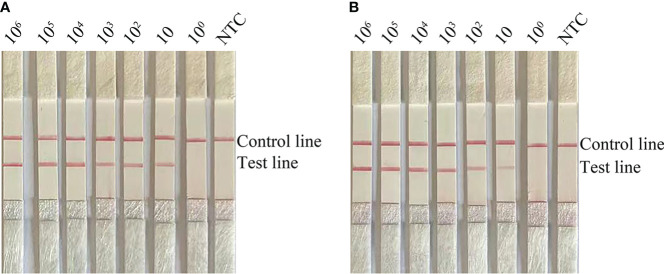
Determination of the LOD of *C glabrata* RPA-LFS. **(A)** The LOD of the *C glabrata* RPA-LFS assay system was established by using primer–probe set F4/R2/P using genomic DNA of *C glabrata* and serial dilutions of 10^0^ to 10^6^ CFU for each reaction. The picture shows the results of RPA-LFS with the number of templates shown at the top of the bar graph. **(B)** The picture shows the results of the RPA-LFS assay using primer–probe set F4/R2/P and 10^5^ CFU of *C albicans* as interference.

To determine the limit of detection (LOD) of the RPA-LFS system for the detection of Candida, 10-fold gradient dilutions of *C. glabrata* ranging from 10^0^ to 10^6^ CFU/µL were tested. In the 50 µL reaction volume, 1 µL of *C. glabrata* genomic DNA was added. A red band appeared on the test line at 10 CFU/µL and became darker as the concentration of *C. glabrata* solution increased (**Figure 2A**). To test whether the system can resist interference from other fungal DNA, 10^5^ CFU/µL of genomic DNA from another common pathogen, *C. albicans*, was added to the RPA reaction. *C. albicans* genomic DNA did not interfere with the detection of *C. glabrata* (**Figure 2B**). The LOD of the RPA-LFS system was 10 CFU/50 µL per reaction. The detection sensitivity was not interfered with by other fungi.

### Interspecies specificity determination of the RPA-LFS assay system

**Figure 3:**
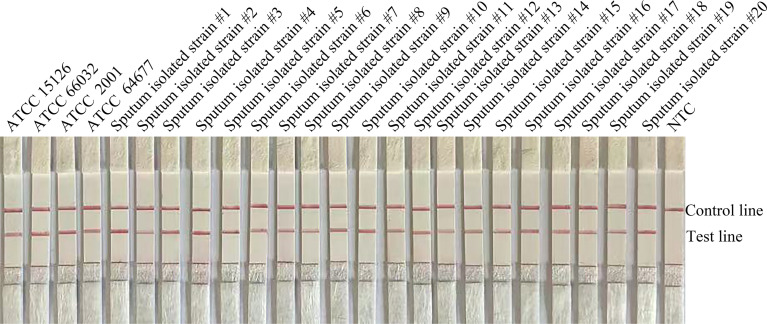
Validation of primer pair F4/R2/P specificity against *C. glabrata*. (A) #1–#20 are the 20 C*. glabrata* isolated from clinical samples. NTC denotes no-template control. The positions of the test and control lines are marked on the right side of the bar graph. Reactions were performed at 37°C for 20 min. Images represent the results of three independent experiments.

**Figure 4:**
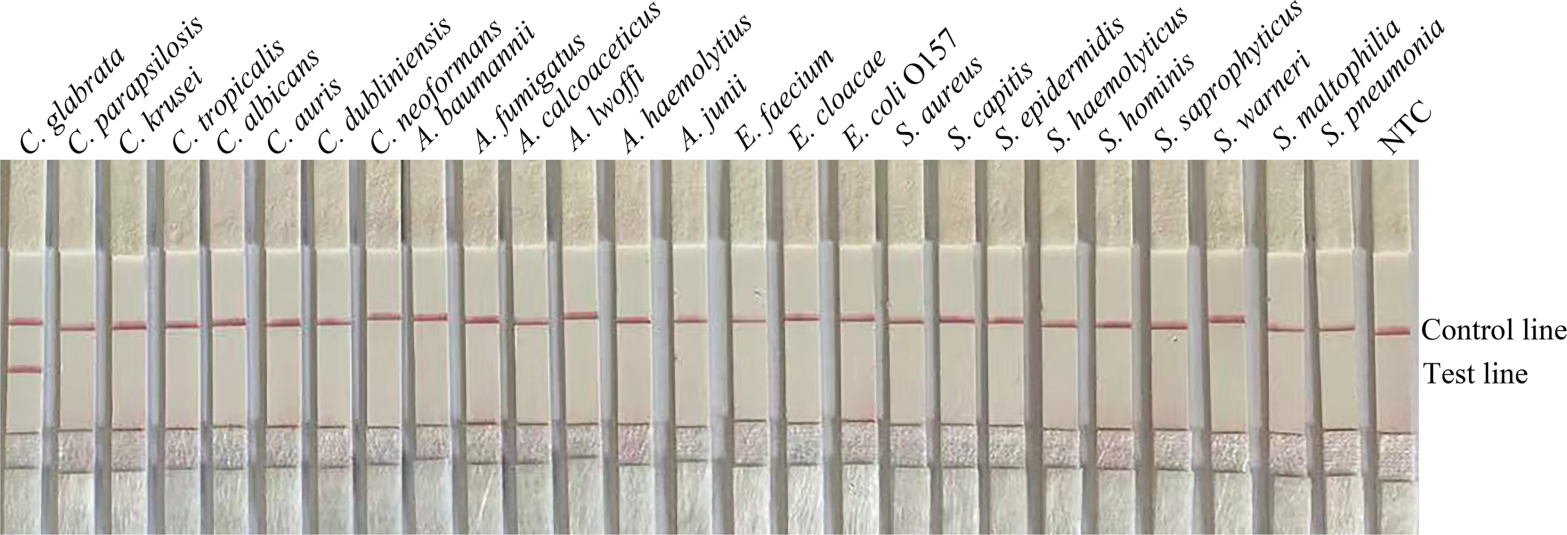
Specificity of F4/R2/P. *C. glabrata* ATCC 15126 was used as a positive control against other pathogenic bacteria tested, and species names are indicated at the top of each strip. NTC indicates no-template control. The positions of the test and control lines are marked on the right side of the bars. Reactions were performed at 37°C for 20 min. Images represent the results of three independent experiments.

To confirm the inclusivity and specificity of F6/R2/P, four reference strains, 20 clinical isolates, and other pathogenic bacteria were amplified with RPA-LFS (**Table 2**). Four reference strains and 20 clinical isolates were positive (**Figure 3**), and the other pathogenic cultures were negative (**Figure 4**). The findings indicated that the primer–probe set has good inclusion and specificity for *C. glabrata* and can effectively detect *C. glabrata*, with no cross-reactivity with other pathogenic bacteria.

**Table 2:** Bacterial strains used in this study.

Species	Source	Strain designation
*C. glabrata*	Reference strain	ATCC 15126
*C. glabrata*	Reference strain	ATCC 66032
*C. glabrata*	Reference strain	ATCC 2001
*C. glabrata*	Reference strain	ATCC 64677
*C. glabrata*	Sputum isolated strain	#1 #2 #3 #4 #5 #6 #7 #8 #9 #10 #11 #12 #13 #14 #15 #16 #17 #18 #19 #20
*C. parapsilosis*	Reference strain	ATCC 22019
*C. krusei*	Reference strain	ATCC 14243
*C. tropicalis*	Reference strain	ATCC 20962
*C. albicans*	Reference strain	ATCC 10231
*C. auris*	Sputum isolated strain	N/A
*C. dubliniensis*	Sputum isolated strain	N/A
*C. neoformans*	Reference strain	ATCC 14116
*A. baumannii*	Reference strain	ATCC 19606
*A. fumigatus*	Sputum isolated strain	N/A
*A. calcoaceticus*	Sputum isolated strain	N/A
*A. lwoffi*	Sputum isolated strain	N/A
*A. haemolytius*	Sputum isolated strain	N/A
*A. junii*	Sputum isolated strain	N/A
*E. faecium*	Sputum isolated strain	N/A
*E. coli* O157	Sputum isolated strain	N/A
*S. aureus*	Sputum isolated strain	N/A
*S. capitis*	Sputum isolated strain	N/A
*S. epidermidis*	Sputum isolated strain	N/A
*S. haemolyticus*	Sputum isolated strain	N/A
*S. hominis*	Sputum isolated strain	N/A
*S. saprophyticus*	Sputum isolated strain	N/A
*S. warneri*	Sputum isolated strain	N/A
*S. maltophilia*	Sputum isolated strain	N/A

ATCC, American Type Culture Collection.

### Analysis of clinical samples

To verify the practical application of RPA-LFS, 227 clinical samples were collected for RPA-LFS, qPCR, and culture. The results are shown in **Table 3**. Eighty-five samples were identified as *C. glabrata*, representing a detection rate of 37.5%. The results were consistent with qPCR and conventional culture methods. The results showed that RPA-LFS had the same accuracy as qPCR and was consistent with the results of the traditional culture method.

**Table 3:** Prevalence of *ITS2* in 227 clinical isolates of *C. glabrata* with the RPA-LFS and qPCR assays.


	RPA-LFS assay	Positive	Negative	Total
qPCR	Positive	85	0	85
			
	Negative	0	142	142
Total		85	142	227

## Discussion

Acquired immunodeficiency syndrome, organ transplantation, radiotherapy, and chemotherapy cause the body’s resistance to decline. This can increase the risk of opportunistic infection by *Candida* spp., as well as causing or complicating endocarditis, pneumonia, and other serious invasive fungal disease. In recent years, the proportion of Candida among the pathogenic fungi isolated clinically has been increasing in China and elsewhere. The clinical isolation rate of *C. albicans* has decreased, and the infections caused by *C. glabrata* have increased significantly (Hernando-Ortiz et al., 2021).

Among the traditional diagnostic methods of *C. glabrata*, the isolation and culture method is time-consuming and cumbersome. True or pseudofilaments are difficult to resolve in *C. glabrata* by the pathological tissue observation method. The yeast generally exists in the form of budding spores or cells, which are not easy to identify in sections. Thus, diagnosis can be missed, resulting in a low diagnostic rate (Yong et al., 2008; Asadzadeh et al., 2019). Serological methods based on antigen detection can only determine the presence of fungal infections. These methods cannot accurately identify the specific causative fungus, as the cell wall antigens of the cell wall of the pathogenic fungus (mainly dextran, galactose, and mannose) are similar (Sendid et al., 2002).

Molecular detection techniques require the selection of diagnostic amplification targets to effectively detect specific species. Many studies have evaluated various methods for detecting *C. glabrata* infections. We designed a pair of primer probes targeting the *ITS2* gene to detect *C. glabrata*. False-positive results were avoided in this study by appropriate base mismatches to reduce the generation of cross-dimers between primers and probes. We designed two pairs of forward and reverse primers based on the target gene and obtained specific RPA amplification primer pair F2/R2. The 16 bases after forward primer F2 were pulled down as the probe. The probe and downstream primers may form cross-dimers, as predicted by Premier 5.0. Only one base at position 41 of the probe eliminated the dimerization; the LOD of base mismatch was not significantly affected. Subsequently, five forward primers were designed upstream of the probe and combined to obtain the best primer–probe sets. Modifications should be made with care to avoid mismatches at the 3’ end of the primer and probe, as this tends to reduce amplification performance. Although the RPA reaction can tolerate seven base mismatches, fewer base substitutions can maximize the detection performance established by the RPA-LFS system. Base modifications are crucial in laying the foundation for screening suitable primers (Piepenburg et al., 2006; Deng et al., 2020). RPA-LFS can accurately detect *C. glabrata*. The LOD of RPA-LFS was 10 CFU, which is higher than that of qPCR, i.e., 1–10 CFU per reaction (Ogata et al., 2015). In addition, the RPA-LFS method is simple and rapid for detecting *C. glabrata* and can be completed within 30 min. This method requires only an isothermal temperature of 37°C. In contrast, PCR and qPCR require temperature-controlled equipment and have relatively long reaction times. Conventional PCR techniques, including nested PCR, reverse PCR, and others, share the characteristics of rapidity, accuracy, specificity, and sensitivity. However, they require post-electrophoretic analysis, which increases the number of steps and prolongs the detection time (Dudiuk et al., 2014; García-Salazar et al., 2022). The evaluation of clinical samples revealed a very high accuracy of RPA-LFS measurements. Results with different patient samples showed that positive samples from the RPA-LFS assay were comparable to positive samples from the qPCR assay, indicating that the RPA-LFS assay provides an alternative detection method. The main disadvantage with RPA-LFS is the problem of laboratory contamination generated by aerosols. Solving this problem will be challenging and will involve strict partitioning of the spiking, reaction, and test areas.

The foregoing findings demonstrate that the RPA-LFS assay is simple, rapid, accurate, and does not require laboratory facilities. It can be combined with a simple and rapid DNA extraction method for home testing of *C. glabrata* infections (Hernández-Carreón et al., 2021). Timely diagnosis can facilitate early treatment of this fungal infection. The establishment of this rapid diagnostic method provides an effective means for early diagnosis and rapid identification of *C. glabrata* infection, and can be used for epidemiological investigations, disease prediction, and health assessments. It is important for correct clinical diagnosis and treatment, rational drug use, avoiding empirical drug use, and reducing drug-resistant strains.

The rapid clinical visualization molecular diagnosis technique of *C. glabrata* based on RPA-LFS developed in this study can obtain detection results within 30 min, with high specificity, high sensitivity, little dependence on instruments. This method has no need of specially trained laboratory personnel, high operability, and on-site detection, which can meet the conditions of remote areas weak primary hospitals or bedside diagnosis needs. The method should prove to be valuable for the rapid detection of *C. glabrata*.

## Data availability statement

The original contributions presented in the study are included in the article/supplementary material. Further inquiries can be directed to the corresponding authors.

## Author contributions

LW, YW, and KW designed the experiments and wrote the manuscript. LZ and YL collected the clinical samples. KW and LW performed the main experiments. YW analyzed the data. All authors reviewed and approved the final version of the manuscript.

## Funding

This study was supported by grants from the Jiangsu University Clinical Medicine Science and Technology Development Fund Project (grant no. JLY2021088), the Lianyungang City Health Science and Technology Project (grant no. 202122), and the Lianyungang Science and Technology Bureau, Municipal Science and Technology Plan (Social Development) Project (grant no. SF2140).

## Acknowledgments

We thank International Science Editing (http://www.internationalscienceediting.com) for editing this manuscript.

## Conflict of interest

The authors declare that the research was conducted in the absence of any commercial or financial relationships that could be construed as a potential conflict of interest.

## Publisher’s note

All claims expressed in this article are solely those of the authors and do not necessarily represent those of their affiliated organizations, or those of the publisher, the editors and the reviewers. Any product that may be evaluated in this article, or claim that may be made by its manufacturer, is not guaranteed or endorsed by the publisher.

## References

[B1] Al-Tekreeti A. R. A. Al-Halbosiy M. M. F. Dheeb B. I. Hashim A. J. Al-Zuhairi A. F. H. Mohammad F. I. (2018). Molecular identification of clinical candida isolates by simple and randomly amplified polymorphic DNA-PCR. Arabian J. Sci. Eng. 43 (1), 163–170. doi: 10.1007/s13369-017-2762-1

[B2] Asadzadeh M. Alanazi A. F. Ahmad S. Al-Sweih N. Khan Z. (2019). Lack of detection of candida nivariensis and candida bracarensis among 440 clinical candida glabrata sensu lato isolates in Kuwait. PloS One 14 (10), e0223920. doi: 10.1371/journal.pone.0223920 31618264 PMC6795469

[B3] Carinelli S. Kühnemund M. Nilsson M. Pividori M. I. (2017). Yoctomole electrochemical genosensing of Ebola virus cDNA by rolling circle and circle to circle amplification. Biosensors Bioelectronics 93, 65–71. doi: 10.1016/j.bios.2016.09.099 27838201

[B4] Deng J. Li Y. Shi W. Liu R. Ma C. Shi C. (2020). Primer design strategy for denaturation bubble-mediated strand exchange amplification. Anal. Biochem. 593, 113593. doi: 10.1016/j.ab.2020.113593 31978455

[B5] Dudiuk C. Gamarra S. Leonardeli F. Jimenez-Ortigosa C. Vitale R. G. Afeltra J.. (2014). Set of classical PCRs for detection of mutations in candida glabrata FKS genes linked with echinocandin resistance. J. Clin. Microbiol. 52 (7), 2609–2614. doi: 10.1128/jcm.01038-14 24829248 PMC4097693

[B6] Fallahi S. Babaei M. Rostami A. Mirahmadi H. Arab-Mazar Z. Sepahvand A. (2020). Diagnosis of candida albicans: conventional diagnostic methods compared to the loop-mediated isothermal amplification (LAMP) assay. Arch. Microbiol. 202 (2), 275–282. doi: 10.1007/s00203-019-01736-7 31641798

[B7] Ferreira L. Sánchez-Juanes F. Porras-Guerra I. García-García M. I. García-Sánchez J. E. González-Buitrago J. M.. (2011). Microorganisms direct identification from blood culture by matrix-assisted laser desorption/ionization time-of-flight mass spectrometry. Clin. Microbiol. Infection 17 (4), 546–551. doi: 10.1111/j.1469-0691.2010.03257.x 20456452

[B8] García-Salazar E. Acosta-Altamirano G. Betancourt-Cisneros P. Reyes-Montes M. D. R. Rosas-De-Paz E. Duarte-Escalante E.. (2022). Detection and molecular identification of eight candida species in clinical samples by simplex PCR. Microorganisms 10, (2). doi: 10.3390/microorganisms10020374 PMC8880469 35208828

[B9] Guinea J. Mezquita S. Gómez A. Padilla B. Zamora E. Sánchez-Luna M.. (2021). Whole genome sequencing confirms candida albicans and candida parapsilosis microsatellite sporadic and persistent clones causing outbreaks of candidemia in neonates. Med. Mycol 60 (1). doi: 10.1093/mmy/myab068 34718724

[B10] Hernández-Carreón O. Hernández-Howell C. Hernández-Hernández G. Herrera-Basurto M. S. González-Gómez B. E. Gutiérrez-Escobedo G.. (2021). Highly specific and rapid molecular detection of candida glabrata in clinical samples. Braz. J. Microbiol. [publication Braz. Soc. Microbiol 52 (4), 1733–1744. doi: 10.1007/s42770-021-00584-2 PMC8578511 34331680

[B11] Hernando-Ortiz A. Eraso E. Quindós G. Mateo E. (2021). Candidiasis by candida glabrata, candida nivariensis and candida bracarensis in galleria mellonella: Virulence and therapeutic responses to echinocandins. J. fungi (Basel Switzerland) 7 (12). doi: 10.3390/jof7120998 PMC8708380 34946981

[B12] Huang C. Huang P. T. Yao J. Y. Li Z. W. Weng L. B. Guo X. G. (2019). Pooled analysis of nuclear acid sequence-based amplification for rapid diagnosis of mycoplasma pneumoniae infection. J. Clin. Lab. Anal. 33 (5), e22879. doi: 10.1002/jcla.22879 30843291 PMC6595323

[B13] Jeong Y. J. Park K. Kim D. E. (2009). Isothermal DNA amplification *in vitro*: the helicase-dependent amplification system. Cell. Mol. Life Sci. CMLS 66 (20), 3325–3336. doi: 10.1007/s00018-009-0094-3 19629390 PMC11115679

[B14] Khodadadi H. Karimi L. Jalalizand N. Adin H. Mirhendi H. (2017). Utilization of size polymorphism in ITS1 and ITS2 regions for identification of pathogenic yeast species. J. Med. Microbiol. 66 (2), 126–133. doi: 10.1099/jmm.0.000426 28260588

[B15] Krcmery V. Barnes A. J. (2002). Non-albicans candida spp. causing fungaemia: pathogenicity and antifungal resistance. J. Hosp Infect. 50 (4), 243–260. doi: 10.1053/jhin.2001.1151 12014897

[B16] Li L. Redding S. Dongari-Bagtzoglou A. (2007). Candida glabrata: an emerging oral opportunistic pathogen. J. Dent. Res. 86 (3), 204–215. doi: 10.1177/154405910708600304 17314251

[B17] Neji S. Trabelsi H. Hadrich I. Cheikhrouhou F. Sellami H. Makni F.. (2017). Molecular study of the candida parapsilosis complex in sfax, Tunisia. Med. Mycol 55 (2), 137–144. doi: 10.1093/mmy/myw063 27555560

[B18] Ogata K. Matsuda K. Tsuji H. Nomoto K. (2015). Sensitive and rapid RT-qPCR quantification of pathogenic candida species in human blood. J. Microbiol. Methods 117, 128–135. doi: 10.1016/j.mimet.2015.07.021 26232708

[B19] Piepenburg O. Williams C. H. Stemple D. L. Armes N. A. (2006). DNA Detection using recombination proteins. PloS Biol. 4 (7), e204. doi: 10.1371/journal.pbio.0040204 16756388 PMC1475771

[B20] Sendid B. Poirot J. L. Tabouret M. Bonnin A. Caillot D. Camus D.. (2002). Combined detection of mannanaemia and antimannan antibodies as a strategy for the diagnosis of systemic infection caused by pathogenic candida species. J. Med. Microbiol. 51 (5), 433–442. doi: 10.1099/0022-1317-51-5-433 11990496

[B21] Souza A. C. Ferreira R. C. Gonçalves S. S. Quindós G. Eraso E. Bizerra F. C.. (2012). Accurate identification of candida parapsilosis (sensu lato) by use of mitochondrial DNA and real-time PCR. J. Clin. Microbiol. 50 (7), 2310–2314. doi: 10.1128/jcm.00303-12 22535986 PMC3405582

[B22] Tati S. Davidow P. McCall A. Hwang-Wong E. Rojas I. G. Cormack B.. (2016). Candida glabrata binding to candida albicans hyphae enables its development in oropharyngeal candidiasis. PloS Pathog. 12 (3), e1005522. doi: 10.1371/journal.ppat.1005522 27029023 PMC4814137

[B23] Wang F. Ge D. Wang L. Li N. Chen H. Zhang Z.. (2021). Rapid and sensitive recombinase polymerase amplification combined with lateral flow strips for detecting candida albicans. Anal. Biochem. 15 (633), 114428. doi: 10.1016/j.ab.2021.114428 34678249

[B24] Yang Q. Guo W. Liu Y. Zhang Y. Ming R. Yuan Y.. (2021). Novel single primer isothermal amplification method for the visual detection of vibrio parahaemolyticus. Food Analytical Methods 14 (10), 1995–2002. doi: 10.1007/s12161-021-02033-0

[B25] Yong P. V. Chong P. P. Lau L. Y. Yeoh R. S. Jamal F. (2008). Molecular identification of candida orthopsilosis isolated from blood culture. Mycopathologia 165 (2), 81–87. doi: 10.1007/s11046-007-9086-8 18266075

[B26] Zhang M. Li R. Ling L. (2017). Homogenous assay for protein detection based on proximity DNA hybridization and isothermal circular strand displacement amplification reaction. Analytical bioanalytical Chem. 409 (16), 4079–4085. doi: 10.1007/s00216-017-0356-0 28424856

